# Analysis of Erythrocyte Sedimentation Rate Order in Epithelial Ovarian Cancer

**DOI:** 10.7150/jca.82941

**Published:** 2023-07-16

**Authors:** Ngadikun, Heru Pradjatmo, Kuncoro Asih Nugroho, Metamalik Pasala, Prasetyastuti

**Affiliations:** 1 Department of Biochemistry, Faculty of Medicine, Public Health and Nursing, Universitas Gadjah Mada, Yogyakarta, Indonesia.; 2 Department of Obstetrics Gynecology, Faculty of Medicine, Public Health and Nursing, Universitas Gadjah Mada, Yogyakarta, Indonesia.; 3 Department of Physics Education, Faculty of Mathematics and Natural Sciences, Universitas Negeri Yogyakarta, Indonesia.; 4 Master Student of Pharmacology, Faculty of Medicine, Public Health and Nursing, Universitas Gadjah Mada, Yogyakarta, Indonesia.

**Keywords:** biomarkers, EOC, ESRO, NLR, RDW

## Abstract

Erythrocyte sedimentation rate order (ESRO), platelet-lymphocyte ratio (PLR), red cell distribution width (RDW), and neutrophil-lymphocyte ratio (NLR) are hematological parameters that reflect the presence of biomarkers in epithelial ovarian cancer (EOC). This study evaluated the best haematological parameter in order to distinguish between EOC patients and healthy individuals. There were a total of 33 patients with EOC as subjects treated in Dr. Sardjito hospital, Yogyakarta, Indonesia and 32 volunteer subjects as controls. Curves of receiver operation, area under the curve, specificity, and sensitivity were estimated. To demonstrate EOC's presence, the ESRO was better than the hematological parameters of RDW, PLR, and NLR (AUC = 0.9245, 0.9010, 0.8351, and 0.7457, respectively; sensitivity and specificity = 83.3 and 90.6; 88.9 and 87.5; 100.0 and 68.8; and 94.4 and 53.1, respectively) at the cut-off points 1,125, 44.2, 163.2, and 2,551. Hence, ESRO is a better parameter to indicate the presence of EOC compared to RDW, PLR and NLR.

## Introduction

The World Health Organization (WHO) reported that there are 150,000 deaths from epithelial ovarian cancer (EOC) worldwide and there are around 22,000 new cases each year 1. This cancer is the most lethal gynecologic cancer in the world. The condition of EOC is even worse if the patient is not diagnosed at an early stage 2. In Indonesia, EOC is the third cause of death for women, in which 829 cases have been reported 3. The gold standard for EOC is a biopsy that confirms the presence of neoplastic cell growth in the ovary. However, it can cause bleeding and infection due to needle insertion.

The CA125 is a specific biomarker for EOC especially used to monitor therapy. The level increases in the early stadium of EOC 4. This increase is caused by the presence of malignancy in the ovary. The CA125 is a carbohydrate antigen with a negative charge caused by the existence of residues of terminal *sialic acid* 5. Besides CA125, fibrinogen level in the blood increases in patients with malignancy. Fibrinogen plays an important role in the coagulation process 6. The CA125 and fibrinogen contribute to cellular interactions in the blood.

The detection of HE4 resulted in a lower sensitivity compared to CA125. However, it shows higher specificity for malignant compared to benign conditions, such that it can improve the accuracy of diagnosis with CA125 levels and also ultrasound scanning 7. Moreover, lower cases of stage I and II EOC are known in a population at high risk of carriers of germline mutations of breast cancer gene-associated DNA repair (BRCA) in recent screening experiment 8. Nevertheless, the present screening strategy using CA125 speed does not give any contribution to the lowering of overall mortality as demonstrated by the United Kingdom Collaborative Trial of Ovarian Cancer Screening (UKTOCS) program 9. Hence, the search for biomarkers, which can enhance the observation of EOCs is urgently needed.

Erythrocytes stick together and move toward the bottom of the container (sedimentation occurs) when anti-coagulated blood is allowed to stand quietly 10. The process is commonly known as a rouleaux formation. Ethylene Diamine Tetra Acetic Acid (EDTA) is a common anticoagulant. This molecule affects the activity of soluble proteins in plasma so that the occurrence of erythrocyte coagulation can be prevented. The erythrocyte sedimentation rate (ESR) depends on many factors, such as the level of plasma proteins that promote rouleaux formation and subsequently the surface-to-volume ratio favoring erythrocyte sedimentation 11-13.

The Westergren method is a commonly used ESR measurement. This method is done by measuring the boundary between clear plasma and red corpuscles, which is the boundary between clear areas in the blood given anticoagulants (plasma regions) with cloudy areas (corpuscles) after being left on the Westergren pipette for 1 hour. Although ESR assessment has a relatively unsatisfactory specificity and sensitiviy, it is still commonly used, practical, and an effortless test 14,15. The spectrometry method can also be used to determine the ESR value by detecting changes in clarity in the upper part of blood-EDTA in a time series 16.

ESR is a practical and economical test frequently requested in clinical medicine. It measures the depth in which erythrocytes have gone down after an hour in a vertical column of anticoagulated blood because of gravity. In 1921, Westergren introduced the most sufficient method for performing the test. In oncology, a high result of ESR corresponds to a generally deficient prognosis for several types of cancers. In patients with substantial tumors, a sedimentation rate higher than 100 mm per hour demonstrates metastatic disease, but for most tumors, this moderately nonspecific result has been supplanted by more accurate diagnostic tests 17. ESR measurement as a diagnostic tool is often not attractive. The Westergren ESR measurement is a commonly used ESR measurement. This method is carried out by measuring the boundary of the phlogistic zone (Bzp), the boundary between clear plasma and red blood cells, which is the boundary between the clear area in anticoagulated blood (plasma area) and the cloudy area (corpuscles) after being left in Westergreen pipette for 1 hour. In this study, we put forward an alternative concept where we use the order of the ESR or ESR order (ESRO). ESRO is the reaction order of ESR. Order 0 means that the aggregation and disaggregation of erythrocytes do not depend on the number of the erythrocytes. The higher the order, the more types of erythrocytes affects the aggregation and disaggregation. Hence, if we just use ESR, we would not observe any mechanism occurring. To the knowledge of the authors, studies concerning the order of the reactions have not been conducted before. Moreover, using the ESRO makes the sensitivity and specificity to become higher.

One of the simplest, repeatable, and economical tests for EOC patients is the complete blood count (CBC). Moreover, to guide the clinical management of EOC patients who will undergo surgery, several CBC specifications are also correlated with patient survival. Furthermore, by gathering evidence on how inflammation contributes to the development of cancer and the growth of tumors, various serum specifications indicating inflammation, e.g.: neutrophil-lymphocyte ratio (NLR) and the platelet-lymphocyte ratio (PLR), have shown the ability to predict the time duration that patients with cancer, including EOC, can survive. In recent research, certain blood cell markers that are associated to inflammation, e.g.: red cell distribution width (RDW), are linked to how well patients with substantial tumor survive 18.

In this study, we perform curve analysis of receiver operating characteristic (ROC) of ESRO, which we compared with NLR and RDW in EOC. Furthermore, several biomarkers such as CA125 and HE4 that have been used to indicate the presence of EOC are also examined based on articles written by previous researchers.

## Methodology

This study was a cross-sectional study. The EOC patients study group consisted of 33 patients with EOC who were being treated in Dr. Sardjito Hospital, Yogyakarta (group 1). The healthy subjects group consisted of 32 normal subjects as control (group 2). The study was conducted at the Central General Hospital of Dr. Sardjito, Laboratory of Clinical Pathology Public Health and Nursing, Universitas Gadjah Mada (FK-KMK UGM) and Inter-University Center Yogyakarta, Indonesia. The group of EOC patients was diagnosed by a specialist based on histopathological examination and other supporting examinations. The control group consisted of normal subjects who have never had cancer and were not descendants of cancer patients. This study was approved by the Medical and Health Ethics Review Committee, Faculty of Medicine, Public Health and Nursing, UGM, number: KE/FK/888/EC/2015 and all patients gave their consent to participate in the study. The exclusion criteria were patients who had a history of or were suffering from more than one type of cancers or other diseases. Healthy subjects were those with no history of cancer or recent sepsis and inflammation. Healthy subjects also were not descendants of cancer patients. There were no other variables (besides EOC) that affect the four observed parameter expressions.

From each subject, 3.0 mL of venous blood was taken and then inserted into the EDTA tube. 1.0 mL of the blood was taken for ESRO examination, which was performed immediately after the samples were collected. ESRO was defined as the rate order of erythrocyte sedimentation observed every 1 minute for 2 hours. The determination of ESRO was obtained from the reading of the phlogistica zone boundary (Bzp), which was observed using the Westergren method based on the general equation integral method of reaction rate kinetics 19,20.

The rest of the 2 mL was used for routine blood tests to determine RDW, PLR, and NLR, which were defined as the absolute neutrophil count divided by the absolute lymphocyte count. The difference in the value of each parameter in the two groups was analysed using a t-test using programming in the Matrix Laboratory (MATLAB) for Windows 7 (p < 0.01). Accuracy of the diagnostic parameter test used ROC curve, the area under the ROC (AUC) curve, and the standard error (SE). Sensitivity and specificity were obtained from the ROC curve at a certain cut-off point. The accuracy criteria are based on the AUC values, i.e.: 0.9 - 1.0 (very high); 0.8 - 0.9 (high) 0.7 - 0.8 (moderate), 0.6 - 0.7 (weak), and 0.5 - 0.6 (very weak) 21.

## Results

There were 65 subjects who participated in this study. Healthy subjects consisted of 32 women while EOC patients consisted of 33 women. The mean age of healthy subjects was 22 years old, while the mean age of EOC patients was 46 years old. There were 5 patients (15.2%) who never undergone chemotherapy, 20 patients (60.6%) had undergone chemotherapy more than 1 time, and 8 patients (24.2%) patients chemotherapy history were still unknown. Most of the patients had IIIC tumor stage (8 patients or 24.2%), followed by IA, IC, IIC, IV, and IVB with 2 patients each (6.1%). There were 14 patients whose chemotherapy history was unknown.

**Figure 1:**
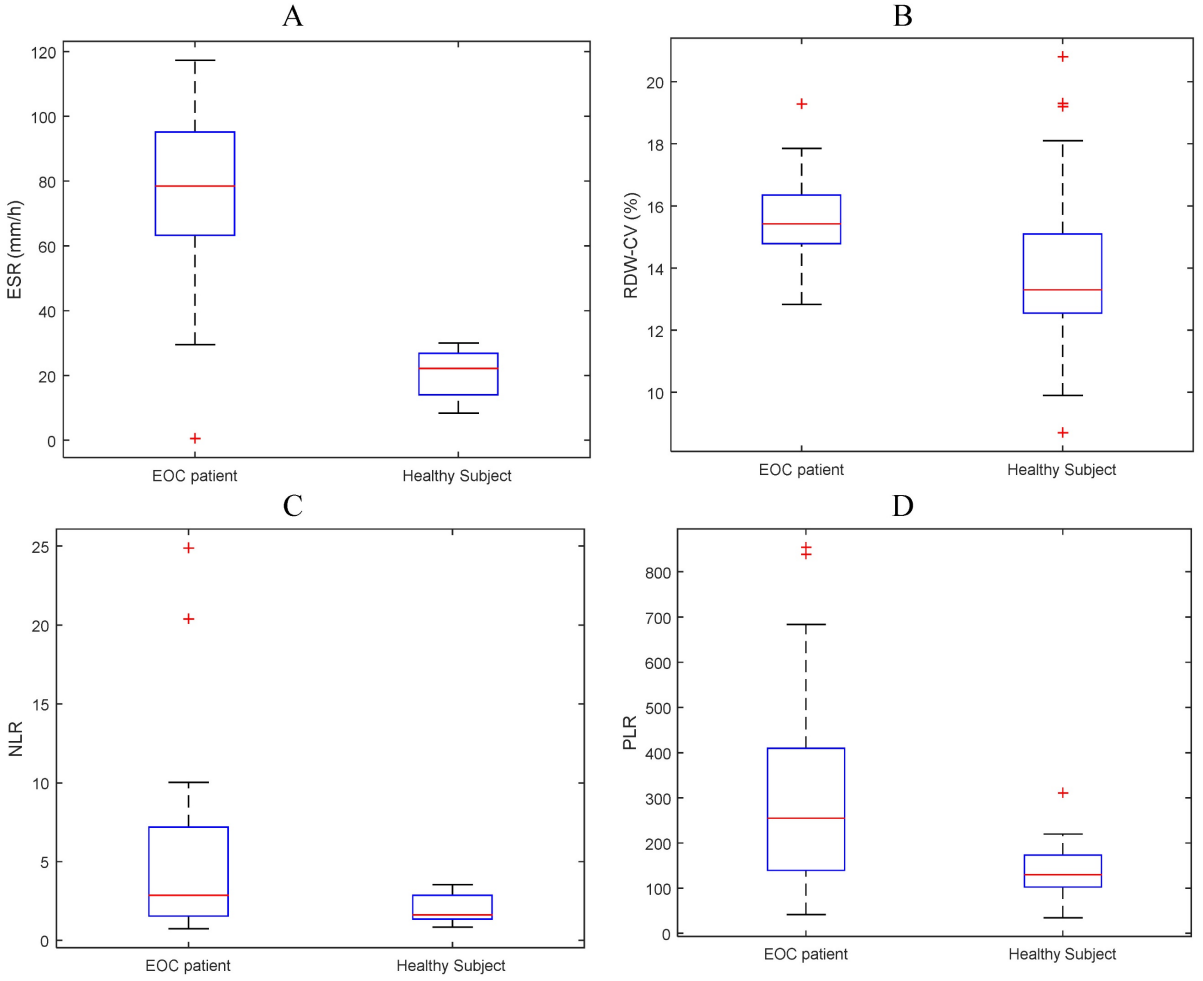
Boxplot of mean **(A)** ESR, **(B)** RDW-CV, **(C)** NLR, and **(D)** PLR for EOC patients and normal subjects.

The mean values ​​of ESR, RDW, NLR and PLR were 4.24, 15.45, 293.44 and 5.13, respectively for the EOC group and 0.18, 14.06, 135.02 and 1.94 respectively for the control group. The mean values ​​of ESR, NLR, and RDW have a tendency for subjects with EOC to be higher than normal subjects (Figure 1 and Table 2).

## Discussion

There is a need to quickly detect EOC to make better prognosis and save women's lives. Therefore, we still need a way to differentiate between pelvic masses in patients with benign gynecological conditions, e.g.: tumors and cysts, and EOC. CA125 is a clinical biomarker to identify EOC. It has limitation as it is affected by menstrual cycle and pregnancy stages. It can also be overdetermined by inflammation and common gynecological conditions, e.g.: endometriosis. Thus, biomarkers that may detect EOC in blood tests must be validated with both harmless and harmful gynecological conditions to make sure they work in real medical situations. In this case, sensitivity is an issue as some EOCs do not show CA125 22. Various studies have discussed many potential biomarkers. However, most of these biomarkers do not meet the requirements for being sensitive and specific enough. In fact, no biomarker outperforms CA125 23,24.

EOC markers in clinical practice are used to predict response to therapy, follow-up after primary therapy, or as prognostic indicators. However, there are currently no recommended blood-borne biomarkers for the diagnosis or screening of EOC. In our study, a new method is introduced to help establish the diagnosis of EOC by measuring changes in ESR using the Westergren method. When the erythrocytes settle, the plasma occupies the top of the blood column. Macromolecules (rouleaux) are formed during the period of aggregation when erythrocytes stick together. Then the cells begin to settle, which can be monitored using this method 15. Within the early stages of cancer, protein levels released as biomarkers are very low. Low level biomarkers cannot be detected using current methods 25. The presence of biomarkers will change the zeta potential of erythrocyte (ZPE) in blood-EDTA 26,27. So that it will affect the balance of erythrocyte aggregation as follows:

nE <===> En (1)

with E is erythrocytes and n is the amount of erythrocytes.

Thus, the presence of biomarkers will affect the Bzp pattern with respect to time. Biomarkers affect the balance of erythrocyte aggregation, when the balance shifts to the right, erythrocyte aggregation occurs which will be followed by sedimentation. Thus, the presence of biomarkers can be analysed by the process of sedimentation.

In this study, the mechanism of deposition was analysed based on the ESRO concept using the integral method. If the rate of aggregation is proportional to the power of the number of erythrocytes, then this can be expressed as 19,20:

R = k(a - x)^n^ (2)

with R is the rate of aggregation; k is the aggregation rate constant; n is the order of occurrence of aggregation (ESRO); a is the number of erythrocytes before the occurrence of aggregation; and x is the number of aggregated erythrocytes. The ESRO value can be obtained by integrating Equation (2) by taking the number of erythrocytes before sedimentation occurs as a, and the number of aggregated erythrocytes as x.

**Figure 2:**
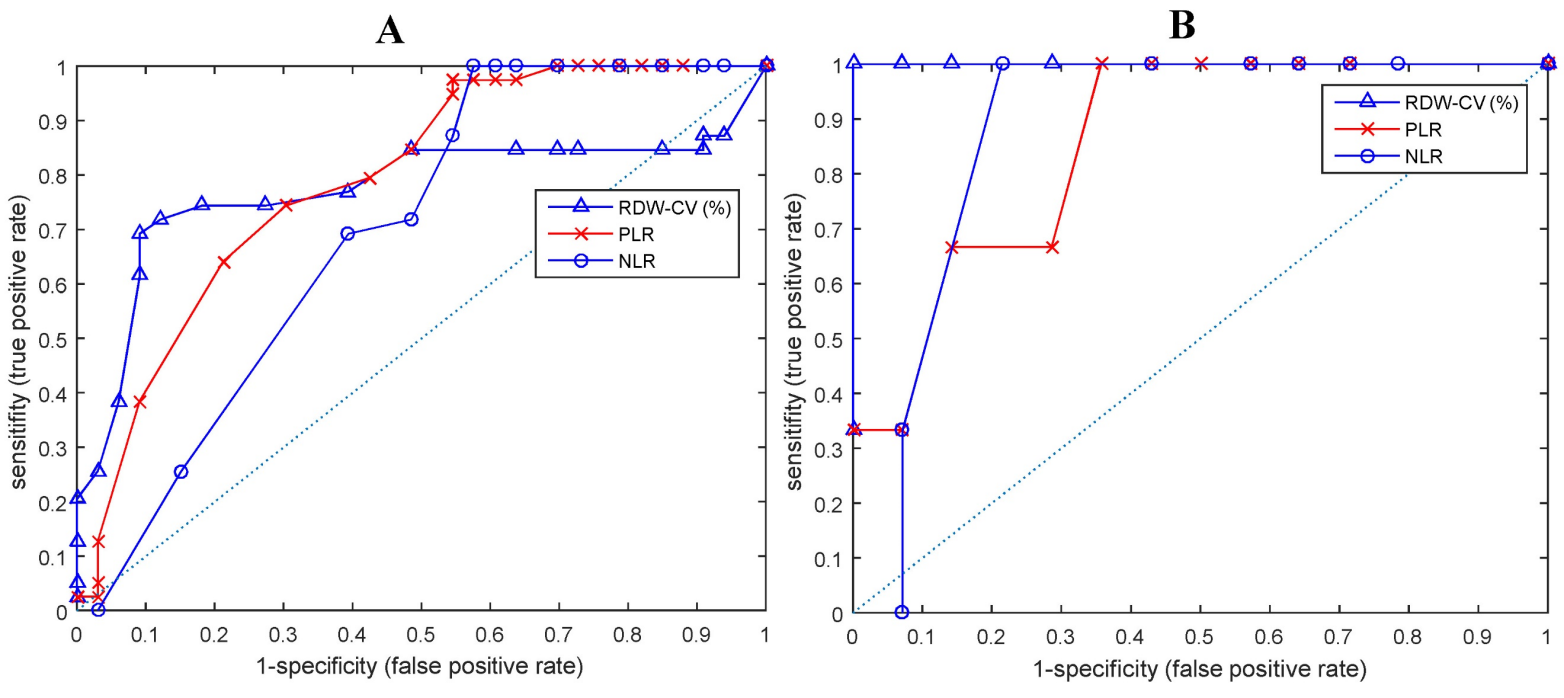
ROC and AUC curves of biomarkers that differentiate between EOC patients and normal subjects in (A) all ESROs, (B) ESRO > 0.

T-test analysis shows that all hematological parameters, i.e.: ESRO, RDW, PLR and NLR could differentiate between EOC patients and normal subjects (p < 0.01). On closer examination, it appears that ESRO can better differentiate between EOC patients and normal subjects (p: 9.16E-07, 3.45E-06, 1.30E-03, and 7.81E-03, respectively - see Figure 2).

**Figure 3:**
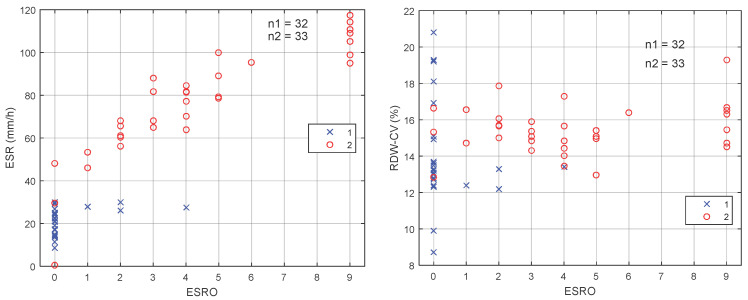
Dimensions scatter diagram of ESRO with (A) ESR and (B) RDW-CV.

Figure 3 shows two dimensions (2D) scatter diagram of ESRO with respect to ESR [Figure 3(A)] and RDW-CV [Figure 3(B)]. It may be observed from the 2D scatter diagram that for ESRO parameter with value of > 0 and ESR > 30 mm/h, the sensitivity and specificity of the ESR are 100%. Hence, by using the ESRO, it is easier to distinguish between EOC and normal patient groups on the RDW, PLR, and NLR parameters.

Scientific evidence suggests that RDW, and other prognostic parameters such as high preoperative NLR, PLR, and MLR predict significantly lower overall survival in all EOC patients, but the mechanisms by which NLR and RDW affect the EOC prognosis is still unclear. However, it has been shown that neutrophils and platelets are connected with in vivo pro-tumor activities, e.g.: increased angiogenesis that contributes to tumor cell proliferation and increases the metastatic potential of tumor cells 28 - 31. Lymphocytes have been considered to play an essential role in guarding against cancer and stopping tumor for growing. The cytotoxic CD8+ T lymphocytes (TIL) are involved in controlling the immune response of EOC. But, not a lot is known about CD8+ TIL prognostic pattern by histotype and how it is related to other medical factors 32.

The sensitivity and specificity for biomarkers of EOC patients with the method we used are greater than those of the current methods. Prior to the onset of cancer, random mutations occur that produce different types of biomarkers, such as CA125 and HE4. Hence, it could be biologically possible that an imbalance in the peripheral neutrophil or platelet to lymphocyte ratio may help understand the underlying tumor growth and prognosis in patients with EOC. This suggests that NLR and RDW can also be used for predictive markers of EOC.

ROC curve analysis shows that ESRO was the most potent diagnostic biomarker for EOC compared to NLR and RDW, with AUC values ​​of 0.8932, compared to 0.7723, 0.7902, and 0.6954, respectively. ESRO also had the highest sensitivity and specificity of 92.3 and 90.9 respectively, at the cut-off points of 0 (Table 3). This shows that the use of ESRO to detect the presence of biomarkers was better than NLR and RDW. In other words, to detect the presence of biomarkers in EOC patients, it was better to use ESRO than RDW, PLR and NLR.

CA125 is the most widely used tumor marker of EOC, although its sensitivity and specificity is far from ideal. This is because its levels are elevated to about 80% of overall EOC and around 50% in first stage of EOC 33. Because of this, the CA125 in not often utilized as the only factor to predict malignancy. Moreover, in order to determine the difference between malignant and benign ovarian masses, the medical history of the patient, results of clinical observation, image data, and tumor biomarker profile are commonly used. Ultrasonography is very helpful in providing the difference between benign and malignant adnexal masses. However, it is also essential to obtain experience and training in correctly differentiating the two adnexal masses 34.

In the past few decades, researchers have been trying to find novel biomarkers for EOC that can be utilized together with or as a substitute for CA125. In 1999, the human epididymal secretory protein 4 (HE4) gene is discovered to be overexpressed in EOC 35. If a cutoff of 12.5% is used for pre-menopausal patients, then the HE4 test produces a sensitivity and specificity of 67.5% and 87.9%, respectively 36. It appears that in term of the ability to distinguish the presence of EOC, ESRO is also better than CA125 and HE4.

The relationship between systemic inflammation and the survival of patients can have important implications for immune treatment in EOC. The connection between inflammation and the spread of cancer suggests that cancer cells that have spread to other parts of the body may be more likely to trigger an immune response and cause more inflammation. ESR is a common test used to check for signs of inflammation in patients. It is found that ESR levels are closely linked to how long EOC patients survived, indicating that inflammation may play a big part in the development of EOC. Some cells that suffer from inflammation may stimulate carcinogenesis by damaging DNA, stimulate tumor cell growth, and promote angiogenesis and metastasis. Thus, the ESRO might provide risk factor and be related to other prognostic parameters. In addition, the ESR assessment is a commonly utilized, affordable, and easy test that might be a good option instead of newer and pricier methods in the prognostic of EOC patients.

Proteins consist of amino acids chains. Different amino acids have different side chains. These side chains affect the acid dissociation constant (pKa) of each of the amino acid. This value describes the ability of amino acids to attract or release H+ ions. The higher the pKa value, the more difficult it is for an amino acid to release H+ ions, and vice versa. The difference in the ability of each amino acid to attract H+ ions, which are positive ions, will certainly affect the movement of ions in the diffuse layer of erythrocytes. The entry and exit of ions in the diffuse layer to the protein will certainly be balanced by the movement of ions from or towards the compact layer. The release of ions from the compact layer to the diffuse layer will affect the erythrocytes zeta potential. An increase in the erythrocytes zeta potential will make the erythrocytes tend to repel each other, and vice versa. There have been several studies examining the important role of the pKa value of protein residues in biological phenomena, one of which was the creation of pKa database (PKAD), which recorded the pKa of 1350 and 232 residues originated from 157 wild-type and 45 mutant proteins, respectively 37. In other words, different proteins certainly have different amino acid residues with different pKa. This has an impact on the tendency of attracting or releasing different ions, which affects the entry and exit of ions in the compact layer of the erythrocytes and leads to adjustment in the erythrocytes zeta potential. These adjustments affect the tendency of erythrocytes to attract or repel each other, which is reflected in the precipitation process that occurs in blood samples, which are observed periodically. Biomarkers themselves are generally proteins that are not expressed in normal cells, so their presence certainly affects the blood settling rate of the sample being observed as previously explained. The sedimentation rate is then expressed as the ESRO.

The novelty in this study is the discovery of a method to determine the presence of biomarkers using only the patient blood samples. The small amounts of biomarkers in early-stage cancer can be identified through this method because the presence of these proteins definitely affects the balance of ions in the blood, as previously explained. This method has been proven to have high specificity and sensitivity compared to other methods that have been commonly used. In addition, this method does not require much cost and special expertise so that its implementation, especially in remote areas, will greatly help cancer detection in its early stage and further improve the success of therapy. Nevertheless, this study had a small number of patients. Research with more patients is needed to confirm the results of this study.

## Conclusion

The findings in this study indicate that ESRO is a better parameter to indicate the presence of EOC than other haematological parameters such as RDW, PLR, and NLR.

## Figures and Tables

**Table 1:** Basic characteristics of the subjects.

No	Study group	Characteristics	Value
1	EOC patients	Number of patients	33
		Age	46 ± 11
		Tumor stage [number (%)]	
		IA	2 (6.1)
		IC	2 (6.1)
		II	1 (3.0)
		IIC	2 (6.1)
		IIIC	8 (24.2)
		IV	2 (6.1)
		IVB	2 (6.1)
		Unknown	14 (42.4)
		Chemotherapy [number (%)]	
		< 1-time	5 (15.2)
		> 1-time	20 (60.6)
		Unknown	8 (24.2)
		Gender [number (%)]	
		Male	0 (0)
		Female	33 (100)
			
			
2	Healthy subjects	Number of healthy subjects	32
		Age	22 ± 4
		Gender [number (%)]	
		Male	0 (0)
		Female	32 (100)

**Table 2:** Comparison of Mean ESR, RDW, PLR, and NLR between EOC patients and normal subjects.

Parameter	EOC		Normal		P value
	mean	SD	mean	SD	
ESR	4.24	2.92	0.18	0.72	3.2E-12
RDW-CV	15.45	1.33	14.06	2.56	6.5E-03
PLR	293.44	215.13	135.02	51.16	3.1E-05
NLR	5.13	5.43	1.94	0.83	5.3E-04

**Table 3:** Sensitivity and Specificity of various haematological parameters.

Parameters	AUC	95% CI		Sensitivity	Specificity	Cutoff value
ESRO	0.8932	0.814	0.9724	92.3	90.9	0
RDW	0.7723	0.6613	0.8834	69.2	90.9	13.8425
PLR	0.7902	0.6828	0.8976	74.4	69.7	157.0995
NLR	0.6954	0.5721	0.8187	100	42.4	3.75726
